# Clinical insights into traumatic injury of the inferior alveolar and lingual nerves: a comprehensive approach from diagnosis to therapeutic interventions

**DOI:** 10.1007/s00784-024-05615-4

**Published:** 2024-03-15

**Authors:** Peer W. Kämmerer, Diana Heimes, Amely Hartmann, Marco Kesting, Fouad Khoury, Eik Schiegnitz, Daniel G. E. Thiem, Jörg Wiltfang, Bilal Al-Nawas, Wolfgang Kämmerer

**Affiliations:** 1 Clinic of Oral and Maxillofacial Surgery, University Medical Center Mainz, Augustusplatz 1, D–55131 Mainz, Germany; 2 Department of Oral and Cranio-Maxillofacial Surgery, Friedrich-Alexander-Universität Erlangen-Nürnberg (FAU), Glückstraße 11, 91054 Erlangen, Germany; 3 International Dental Implant Center, Private Clinic Schloss Schellenstein, Am Schellenstein 1, 59939 Olsberg, Germany; 4 Department of Oral and Maxillofacial Surgery, Christian Albrechts University, UKSH Campus Kiel, 24105 Kiel, Germany; 5 Pharmacy Department, University of Augsburg, Medical Faculty, D–86156 Augsburg, Germany

**Keywords:** Nerve injury, Trauma, Hypoesthesia, Anesthesia, Neuropathic pain, Life quality, Medication, Review

## Abstract

**Objectives:**

This scoping review explores the risk and management of traumatic injuries to the inferior alveolar and lingual nerves during mandibular dental procedures. Emphasizing the significance of diagnostic tools, the review amalgamates existing knowledge to offer a comprehensive overview.

**Materials and methods:**

A literature search across PubMed, Embase, and Cochrane Library informed the analysis.

**Results:**

Traumatic injuries often lead to hypo-/anesthesia and neuropathic pain, impacting individuals psychologically and socially. Diagnosis involves thorough anamnesis, clinical-neurological evaluations, and radiographic imaging. Severity varies, allowing for conservative or surgical interventions. Immediate action is recommended for reversible causes, while surgical therapies like decompression, readaptation, or reconstruction yield favorable outcomes. Conservative management, utilizing topical anesthesia, capsaicin, and systemic medications (tricyclic antidepressants, antipsychotics, and serotonin-norepinephrine-reuptake-inhibitors), proves effective for neuropathic pain.

**Conclusions:**

Traumatic nerve injuries, though common in dental surgery, often go unrecorded. Despite lacking a definitive diagnostic gold standard, a meticulous examination of the injury and subsequent impairments is crucial.

**Clinical relevance:**

Tailoring treatment to each case's characteristics is essential, recognizing the absence of a universal solution. This approach aims to optimize outcomes, restore functionality, and improve the quality of life for affected individuals.

**Graphical abstract:**

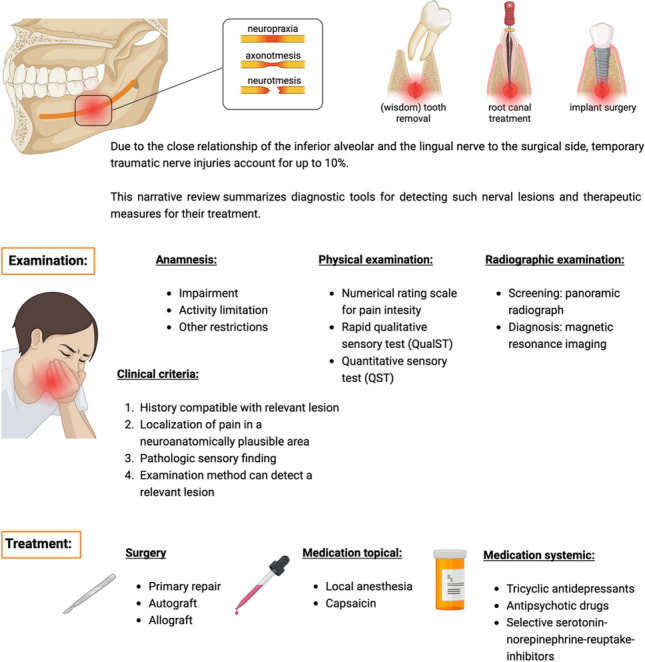

## Introduction

The osteotomy procedure for impacted wisdom teeth ranks among the most commonly executed dental surgical interventions on a global scale. The intricate anatomical proximity during wisdom tooth removal gives rise to reported injury rates of 0.4–5.5% for the inferior alveolar nerve and 0.06–10% for the lingual nerve. Notably, the incidence of persistent sensory impairment following these surgical interventions has been estimated to range between 0.4% and 13% [1]. In addition to surgical tooth extraction, other prevalent detrimental procedures include the inadvertent displacement of root filling material into the mandibular canal, administration of local anesthesia, maxillofacial trauma, and the insertion of dental implants [1–4]. Studies suggest that merely visualizing the neurovascular bundle during surgery is associated with a noteworthy 20% risk of postoperative paresthesia [5].

The precise incidence of nerve injuries remains elusive due to the likely significant number of unreported cases. The prevalence of (temporary) nerve injuries varies across specific dental procedures, with reported rates of 0.15% for surgeries like cystectomies or block anesthesia of the inferior alveolar nerve [6], 8–12% for wisdom tooth removal [7–9], exceeding 30% in dental implantology [10, 11], and reaching up to 60% in orthognathic surgery [12, 13]. Nevertheless, permanent nerve injuries occur with considerably less frequency. It is theorized that persistent impairment of trigeminal branches, characterized by symptoms lasting beyond three to six months, may be associated with up to 1% of all dental, oral, and maxillofacial surgical procedures [6]. The likelihood of persistent nerve function impairment increases in cases where there is discernible evidence of severe nerve damage resulting from surgery, elevated patient age at the time of the injury, and when the damage is located in closer proximity to the main nerve [14, 15]. Compression injuries can lead to impaired sensory perception in the affected areas of the face. Chemodenervation, often associated with certain toxins including the local anesthetic solution, disrupts nerve signaling and can result in temporary or permanently altered sensation. Transection injuries, typically caused by trauma or surgical intervention, sever the nerve fibers completely, leading to significant sensory deficits in the affected regions [16]. In accordance with Seddon's classification [17], nerve injuries can be categorized into three main types: 1) neuropraxia, characterized by a temporary interruption of conduction without loss of axonal continuity, often attributed to stretching or pressure; 2) axonotmesis, involving the loss of both axonal continuity and its myelin covering, while preserving the connective tissue framework of the nerve; and 3) neurotmesis, a complete disruption of the entire nerve fiber.

Patients frequently experience not only primary sensory deficits, such as anesthesia, hypoesthesia, or paresthesia, but also endure neuropathic pain characterized by dysesthesia, allodynia, or hyperalgesia. Neuropathic pain, defined as pain ensuing from an injury to the somatosensory system, adds a complex dimension to the clinical manifestation of nerve injuries [18, 19]. In addition to the positive sensory symptoms, the sensory deficit associated with the inferior alveolar nerve may give rise to functional disturbances, including uncontrolled salivation, lip biting, and speech difficulties. Furthermore, injury to the lingual nerve can be correlated with additional complications such as taste loss, tongue biting, difficulty in articulation, and challenges in controlling food during eating [20]. These complaints are often linked to a diminished quality of life, imposing notable psychological and social limitations on affected individuals [21].

As of now, a systematic guideline for diagnosing and treating traumatic injuries to the inferior alveolar and lingual nerves following dental surgery does not exist. Consequently, this scoping review seeks to distill insights from international literature, with the objective of formulating guidelines to assist dental practitioners in the diagnosis and treatment of such injuries.

The first subsection provides a comprehensive review of the existing literature pertaining to the diagnosis of traumatic nerve injuries. It explores various aspects of traumatic nerve injury diagnosis, including clinical assessments, imaging techniques, and emerging trends in the literature. The second subsection covers conservative and surgical treatments, rehabilitation strategies, and potential innovative, providing a comprehensive overview of the current landscape in traumatic nerve injury therapeutics.

## Methods

The research question was formulated, prompting a systematic search across electronic databases such as PubMed, Embase, and the Cochrane Library. Additionally, manual searches were conducted for national and international guidelines. The search terms encompassed key phrases like "nerve injury/damage," "anesthesia," "hypoesthesia," "paresthesia," "neuropathic pain," "diagnosis/diagnostic," "examination," "treatment," "medication," "surgery," and "conservative." Reference lists of retrieved articles were scrutinized for additional relevant studies. A compilation of pertinent studies was created, with titles and abstracts screened against the criteria shown above. Subsequently, relevant data from the included studies were systematically extracted, covering study design, sample size, intervention, outcome measures, and results. Upon analysis of the extracted data, discernible patterns and trends were identified. In the final stage, conclusions were descriptively drawn based on the summarized results, providing insights into the diagnosis and treatment of traumatic nerve injuries in the orofacial region.

## Results and discussion

### Diagnosis of traumatic nerve injury

Accurate assessment of traumatic nerve injuries necessitates more than a simple mechano-sensory evaluation. Drawing inspiration from World Health Organization (WHO) guidelines, a comprehensive examination of the injury should encompass considerations of impairments, activity limitations, and other associated restrictions [14, 15]. Diagnosing and treating traumatic damage to trigeminal branches presents inherent challenges, leading to potential issues such as delayed referral to a specialist, instances of both over- and undertreatment, substantial costs to the healthcare system, and the emergence of legal claims. During the initial presentation of an affected patient, it is crucial to distinguish between certain, probable, possible, and unlikely nerve injuries. In particular, Schlereth et al. propose specific criteria for assessing neuropathic pain in detail [18, 19]:

1. the patient's history aligns with a pertinent lesion,
2. the pain is localized in a neuroanatomically plausible area,
3. pathologic sensory findings are present within the neuroanatomically plausible area of pain spread and
4. at least one examination method can identify a relevant lesion in the peripheral somatosensory system.

**Fig. 1:**
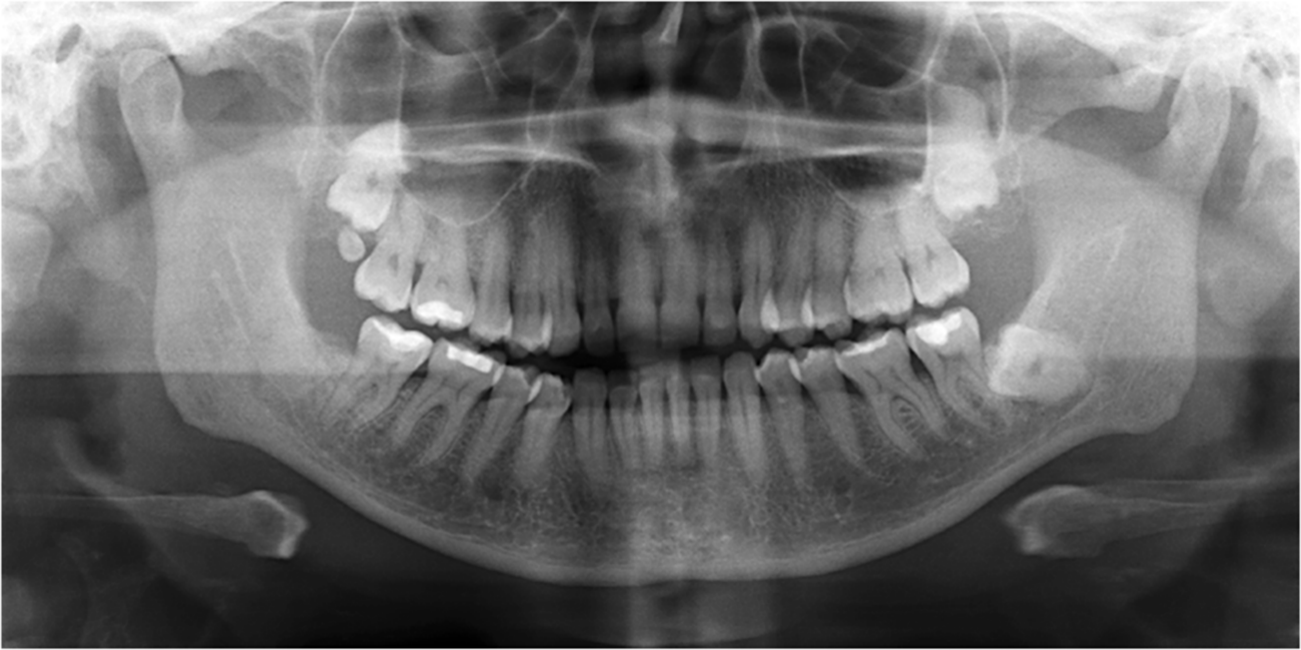
Panoramic radiograph depicting a patient presenting with numbness of the right lip and chin. The removal of the lower wisdom tooth on the corresponding side occurred one year ago. The radiograph clearly displays the lower border of the nerve canal of the inferior alveolar nerve, with a somewhat blurred upper border. Notably, a tooth-shaped dense region is evident, overlapping the nerve canal, raising concerns about a potential injury to the inferior alveolar nerve

While there is no definitive gold standard for diagnosing nerve injury and neuropathic pain, it is advisable to conduct a comprehensive medical history and clinical examination. The assessment should encompass details regarding the onset, duration, time course, character, location, severity, and any precipitating or alleviating factors associated with the complaints. Additionally, information pertaining to functional impairments, consequences of the lesions, and any prior treatments, whether successful or unsuccessful, should be considered. It is crucial not to overlook comorbidities, including anxiety, depression, and/or sleep disorders, which often necessitate interdisciplinary patient care [18]. Moreover, it is essential to visualize the local anatomical conditions through appropriate radiographic imaging. A panoramic radiograph may be selected as a screening tool to assess the patient and eliminate other potential causes for the symptoms (Fig. 1).

**Fig. 2:**
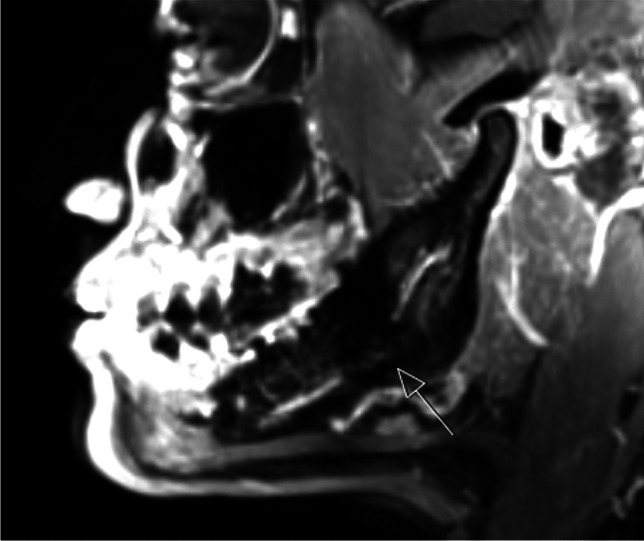
Magnetic resonance imaging of the right lower jaw from the patient depicted in Fig. 1, shown in a sagittal section. The nerve, presented as a light-shaded hyperintense structure, is clearly discernible within the darker, hypointense bone structure. The arrow highlights a conspicuous interruption in the structure of the inferior alveolar nerve precisely at the location of the prior wisdom tooth osteotomy. This observation significantly aids in the diagnosis of nerve damage

To provide a detailed depiction of the nerve anatomy, magnetic resonance imaging (MRI) is generally recommended (Fig. 2) [22, 23]. Conventional radiographs solely capture the osseous boundaries of the mandibular canal. In contrast, MRI, utilizing specialized sequences, can concurrently visualize both osseous structures and neural tissue within the oral cavity [24].

In the initiation and progression of therapy, analog scales have demonstrated utility in quantifying pain intensity. The 11-step numerical rating scale (NRS; 0 = no pain, 10 = maximum imaginable pain) and various visual analog scales are especially effective in this context. With these scales, the patient indicates their pain intensity by marking a point on an unmarked horizontal line. Likert scales, featuring descriptors and icons, offer an alternative, particularly well-suited for children, foreign language speakers, and cognitively impaired patients. These scales enable a rapid and tangible representation of subjective improvements or deteriorations over time.

To enhance diagnostic certainty, the utilization of a rapid qualitative sensory test (QualST) as a bedside assessment and/or a more comprehensive quantitative sensory test (QST) is recommended, in accordance with the recommendations of the German Neuropathic Pain Research Network (DFNS) [19, 25]. The German guideline on analogous diagnosis and non-interventional therapy of neuropathic pain mandates the confirmation of damage to the somatosensory system through clinical-neurological examination and instrumental diagnostics [18].

The QualST can be conducted using instruments readily available in any office or clinic. This test procedure evaluates bilateral tactile stimuli, pinpricks, and thermal stimuli to document the relative differences between the two sides [21, 26]. The QST is a non-invasive psychophysical testing method employed for the assessment, diagnosis, and monitoring of somatosensory deficits and sensory neuropathies [27, 28]. QST employs calibrated specific test algorithms to objectively measure subjects' responses to harmless or graded harmful thermal and/or mechanical stimuli. This method is valuable in distinguishing whether nerve injury has an inflammatory origin or if there is permanent nerve damage. Neuropathic pain resulting from inflammation may be associated with a decrease in the detection threshold of the affected nerve, indicating a higher likelihood of reversibility. Conversely, increased detection thresholds are often observed in cases of permanent nerve damage [29]. Nevertheless, QST is a time-consuming process that demands costly specialized equipment and well-trained, calibrated investigators to achieve standardized and reproducible results [21, 30, 31]. Moreover, the use of validated questionnaires is recommended for screening or assessing the severity of neuropathy. Scales employed in this context typically encompass the typical pain characteristics, covering both positive and negative symptoms. These may include parameters such as pain intensity, localization, and the radiation of the symptoms [18].

## Treatment of traumatic nerve injury

### Patient education

Given the elective nature of many dental procedures and patients' anticipation of functional or aesthetic enhancement, coping with the consequences of traumatic or iatrogenic nerve damage can be challenging. This is exacerbated, particularly in cases where preoperative patient education has been inadequate. Identification of any nerve damage should be promptly conducted within the initial 24 h postoperatively. It's noteworthy that damage to the inferior alveolar and lingual nerves may be masked by nerve block anesthesia, underscoring the importance of vigilant postoperative monitoring [32]. In the event of a nerve injury, it is crucial to inform the patient about the anticipated duration of the symptoms. Open and honest communication not only fosters trust but also enables timely and efficient treatment [33]. Especially in cases where a peripheral nerve is entirely severed, patients should be apprised of the potential permanence of the lesion. It is imperative to emphasize the immediate necessity of consulting with an oral and maxillofacial reconstructive surgeon for possible reconstruction. Clear communication in such scenarios is essential to guide patients toward appropriate and timely interventions [15].

### Treatment timing and strategies

**Fig. 3:**
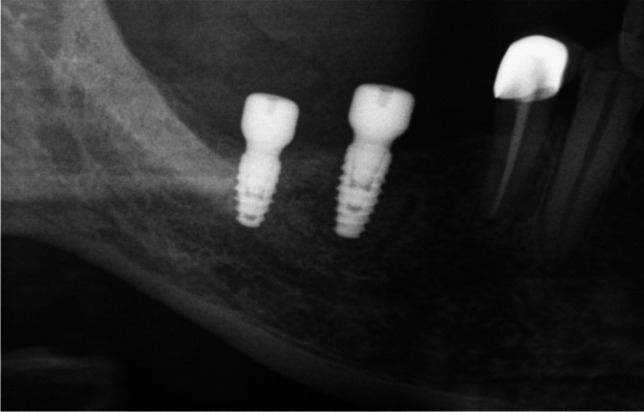
Section of a panoramic radiograph illustrating a dental implant in the region of tooth 47, demonstrating its close proximity to the inferior alveolar nerve. The placement of the implant resulted in a mild hyposthesia experienced by the patient in the chin region

**Fig. 4:**
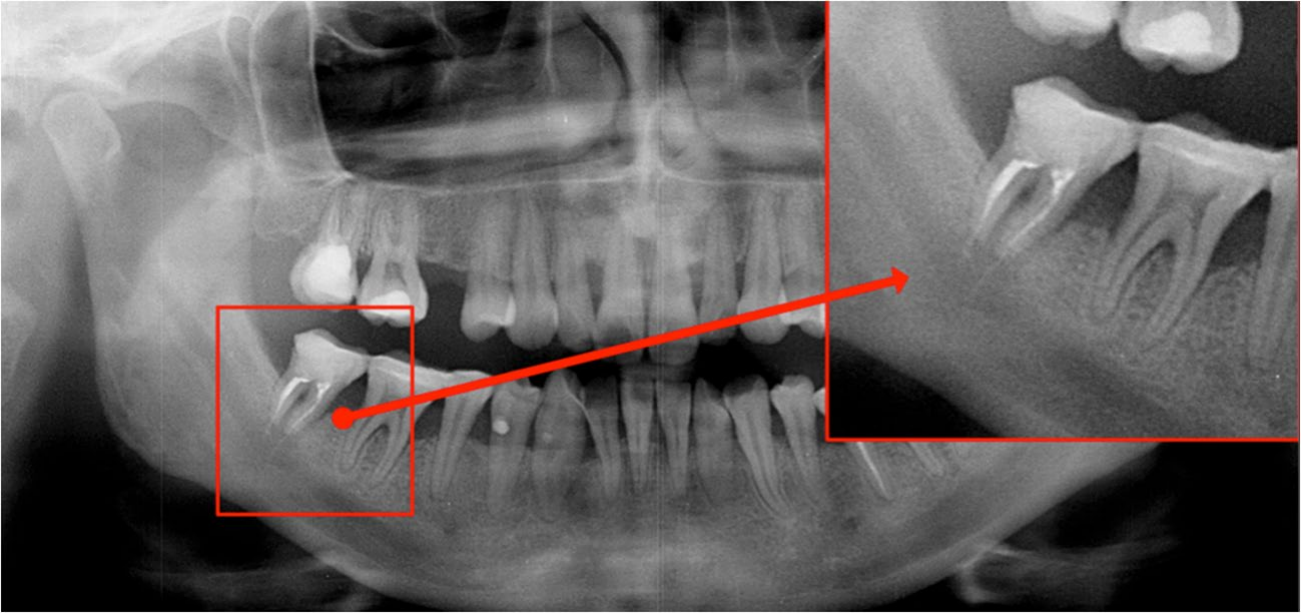
Panoramic radiograph of a patient where root filling material was accidentally overfilled into the nerve canal in the mesial root of tooth 47. This resulted in recurrent neuropathic pain, which disappeared after the removal of the material

Nerve injuries can be addressed through conservative or surgical approaches. Immediate repair at the moment of injury is generally recommended, as it ensures the best functional outcomes. Early intervention can significantly contribute to optimal recovery and functional restoration [34]. In cases where nerve damage is attributed to a foreign body, such as a dental implant (Fig. 3) or an overstuffed root canal filling (Fig. 4), prompt action is essential. Ideally, the foreign body should be removed within the first 36 h after insertion to mitigate potential complications and enhance the prospects of nerve recovery. Swift intervention in such scenarios is crucial for minimizing adverse effects and optimizing the chances of successful resolution [1, 35, 36].

Given the diverse clinical manifestations, ranging from hypoesthesia to anesthesia, and the presence of bothersome symptoms such as paresthesia, dysesthesia, and hyperalgesia, there lacks a universal and standardized therapeutic approach. The methods of treatment, the optimal timing for conservative or surgical therapy, and the selection of medication or biomaterial are subjects of contentious debate. The spectrum of options spans from a "wait-and-see" approach to early or delayed surgical interventions, along with conservative medical treatments. The lack of consensus underscores the complexity of addressing traumatic nerve injuries, necessitating individualized and carefully considered approaches based on the unique characteristics of each case [37, 38]. Treatment for traumatic nerve injuries may encounter delays due to misinterpretation, often perceived as prolonged local anesthesia or the need for referral to another surgeon. Such delays may extend over several days or weeks, impacting the timely initiation of appropriate interventions. Nerve regeneration occurs at a relatively slow pace, influenced by factors such as patient age, the extent of injury, and the presence of underlying medical conditions. Additionally, the accessibility of denervated motor-end-plates, particularly in cases of prolonged nerve injury, may impact the effectiveness of surgical interventions aimed at restoring function [16]. Additionally, patient- and practitioner-related factors, including general health conditions, patient compliance, and the practitioner's skills and knowledge, can influence the choice of treatment. Recognizing and addressing these factors is essential for optimizing the overall treatment outcome and patient experience. In cases where nerve damage has persisted for an extended duration, conservative treatment may be considered as the method of choice. Nonetheless, certain authors advocate for exploratory surgery if there is no improvement in nerve function after 3–6 months. The decision between conservative management and surgical exploration should be based on careful evaluation of the specific circumstances and individual patient response, with the goal of optimizing outcomes and ensuring the most suitable course of action [14, 39, 40]. Divergent findings in the literature suggest varying perspectives on the optimal timing for nerve reconstruction after injury. Some research groups highlight a significantly increased risk of permanent damage if reconstruction is delayed beyond 9 [41] or 12 months [42]. The temporal aspect after surgery emerges as a critical factor, with certain authors noting substantially improved recovery rates 12 months post-injury compared to shorter follow-up periods [43–45]. The significance of the time elapsed after nerve injury in determining treatment success remains inconclusive. While some authors contend that an interval exceeding ten weeks between injury and surgery may be too long [46], others have reported very good success rates for nerve grafting even after more extended intervals. The variability in findings underscores the complexity of factors influencing treatment outcomes, emphasizing the need for individualized assessments and considerations in the management of traumatic nerve injuries [47, 48].

Psychological symptoms often accompany neuropathic pain, highlighting the importance of a multidisciplinary approach to treatment. In addition to the conservative and surgical options for treating traumatic injury to the trigeminal nerve or its branches outlined below, pain psychotherapy can be a crucial component. However, it is essential to note that the data in this area need improvement to make evidence-based statements. Enhancing the understanding of the interplay between psychological factors and neuropathic pain can contribute to the development of more effective and holistic treatment strategies [49, 50].

### Surgical treatment

#### Primary nerve repair

**Fig. 5:**
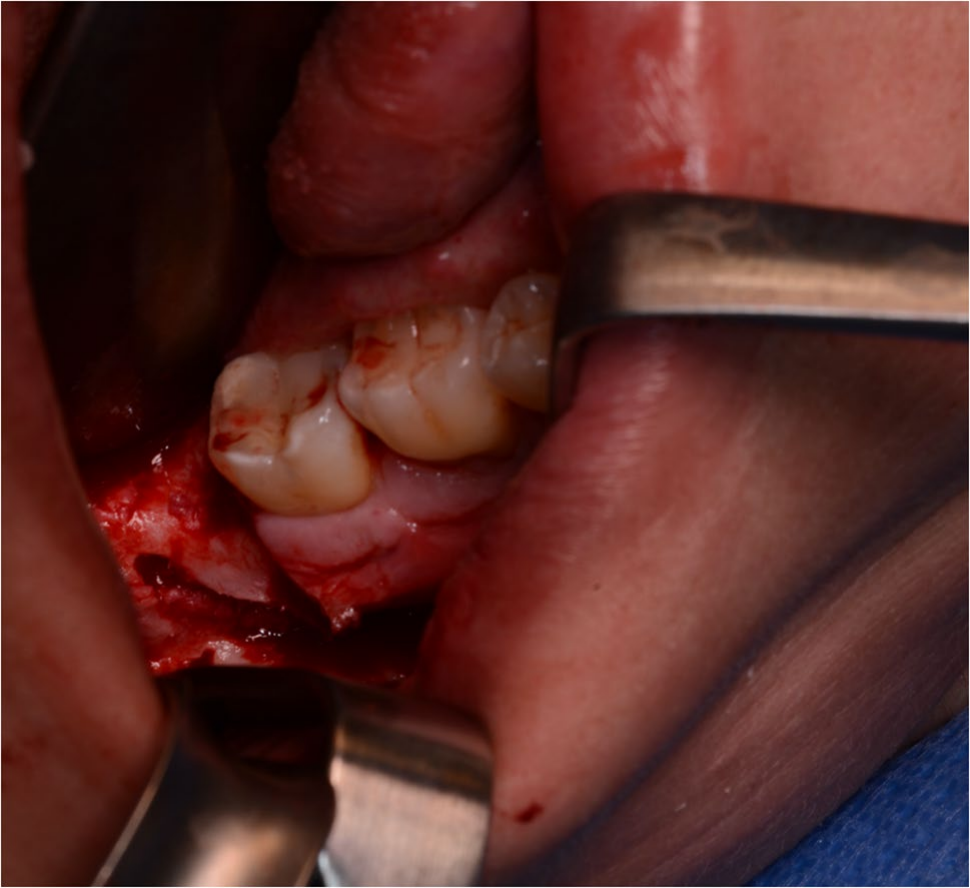
Clinical image depicting the surgical decompression of the right inferior alveolar nerve, which sustained damage during wisdom tooth osteotomy

In cases where nerve compression is suspected, such as after wisdom tooth removal, placement of dental implants, or endodontic treatments, timely surgical intervention is recommended. Ideally, this intervention should be performed within the first 24–36 h to address the compression and mitigate potential complications. Swift action in such instances is crucial for optimizing outcomes and preventing prolonged nerve damage [14]. In such cases, surgical options typically involve either the removal of external compression on the inferior alveolar nerve or surgical decompression of the injured nerve itself (Fig. 5).

Studies have demonstrated that after decompression, sensory function was restored in a notable percentage of cases, ranging from 85 to 100%. This highlights the efficacy of surgical interventions, particularly decompression, in addressing nerve injuries and promoting sensory recovery [51, 52]. Consistent with these findings, other authors have reported that decompression alone led to faster recovery compared to alternative nerve repair procedures [53].

For cases involving complete neurotmesis (transection of the nerve) or neuroma formation [54], where conservative treatments or simple decompression may be less promising, alternative surgical interventions are recommended [55]. Thus, two primary surgical options are available: direct nerve anastomoses and reconstructions using (autologous) grafts.

**Fig. 6:**
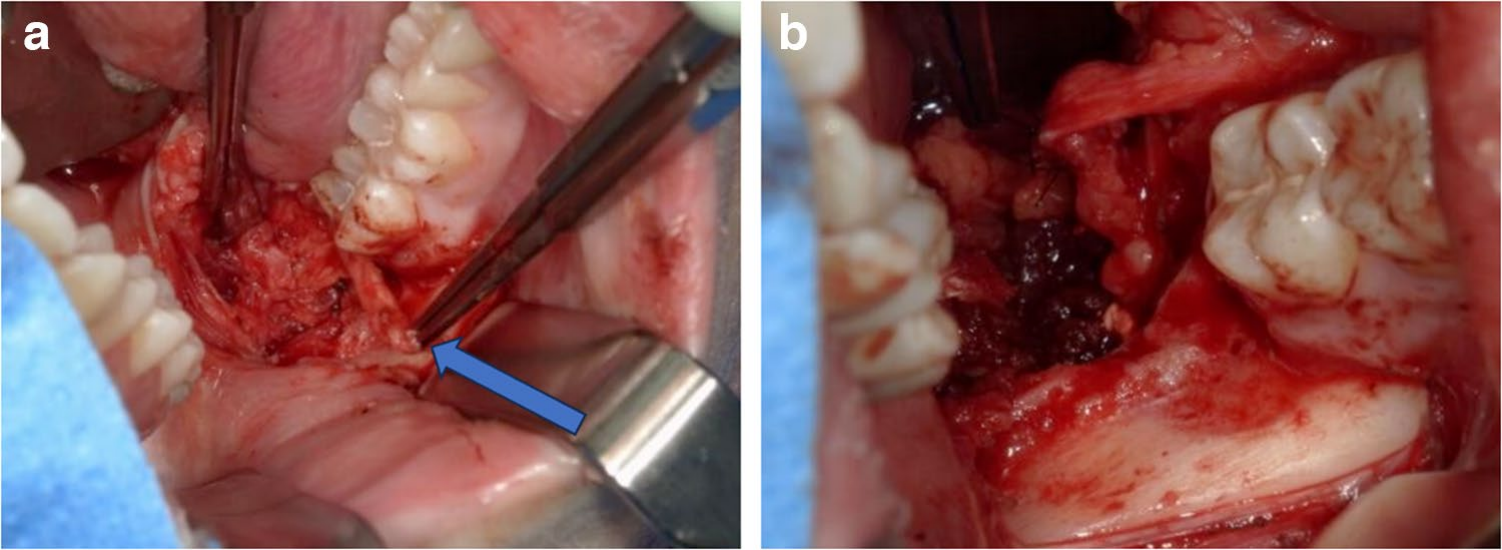
Clinical site presentation following impaction of teeth 48 with iatrogenic rupture of the lingual nerve. In Fig. 6a, the lingual nerve's iatrogenic rupture is visually indicated by a distinguished blue arrow. Moving forward, Fig. 6b showcases the repair of the affected nerve through a direct anastomosis procedure employing 8–0 sutures

Whenever feasible, a tension-free nerve suture should be prioritized as the preferred option [56] (see Fig. 6a and b).

The success of this procedure relies on precise adjustment by approximating the pedicles for regeneration, a condition that is typically achievable in defects less than 1 cm in size [38]. Direct suturing presents advantages due to the limited length of regeneration required and the necessity for only one anastomosis. Despite its technical sensitivity, this approach is beneficial in minimizing complexity while optimizing the potential for successful nerve repair. If the fascicles are not adequately aligned or the nerve undergoes trauma due to improper suture placement, the result may be uneven regeneration, and successful and complete recovery might not be assured. However, with careful technique and under optimal circumstances, the likelihood of achieving sensory recovery can be as high as 90% [51]. Primary reconstruction is recommended within two weeks after injury and is preferred over secondary or delayed nerve repair if specific conditions are met. Successful primary reconstruction necessitates a sharp incision of the injured nerve without bruising, a clean wound bed, and the absence of associated injuries. Additionally, the patient must be eligible for surgery under intubation anesthesia [57].

Early secondary reconstruction involves a planned second intervention within a few weeks of initial healing. While this approach provides an opportunity for further intervention, it comes with the potential drawback of elastic retraction of the nerve during initial healing, making direct coadaptation challenging. Later secondary reconstruction is employed when primary or early secondary attempts have proven unsuccessful or when initial nerve repair was not conducted. The extended delay in treatment under these circumstances creates less favorable conditions, resulting in a prognosis that is worse and, therefore, not comparable to that of earlier interventions [58].

#### Autografts

**Fig. 7:**
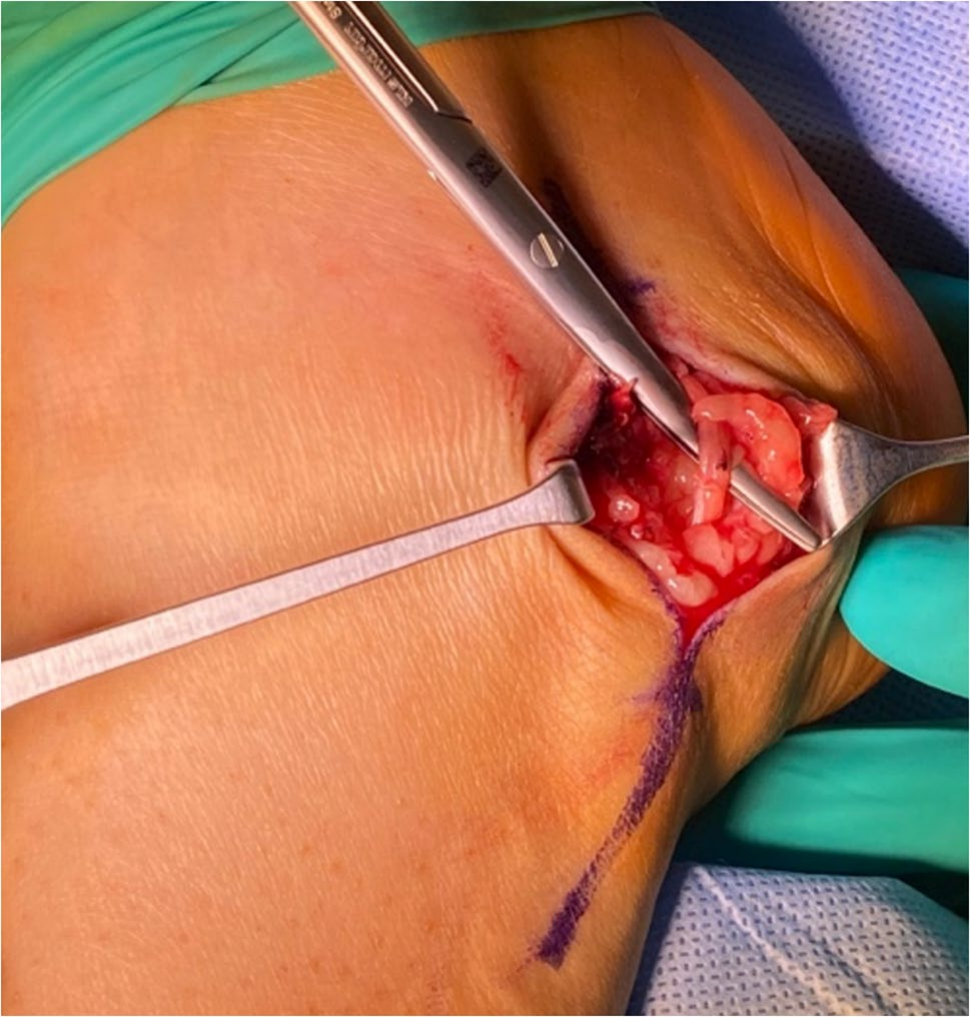
Sural nerve removal. The sural nerve typically offers cutaneous innervation to the skin of the posterior to posterolateral leg and can be harvested at the ankle level with minimal postoperative morbidity

In instances where the primary adaptation technique is unfeasible, the prevailing therapeutic gold standard involves the utilization of an autologous nerve graft. The decision regarding treatment should be predicated upon an assessment of the gap's length and the specific nature of the nerve injury, with the overarching objective of achieving a tension-free repair. An autologous nerve graft serves as a conduit, supplying neurotrophic factors and Schwann cells, while concurrently acting as a scaffold to facilitate nerve regeneration. Typically, donor tissue for this procedure is sourced from the sural nerve (see Fig. 7 [59]) and/or the auricular magnus nerve.

The sural nerve is frequently selected for grafting due to its well-matched diameter and fascicular pattern, aligning closely with the characteristics of the trigeminal nerve. This choice is driven by the optimal compatibility between the two nerves, enhancing the likelihood of successful graft integration and functional recovery [60]. The literature consistently reports a noteworthy nerve restoration rate, ranging from 87 to 100%, when employing autologous nerve grafts [51, 61]. While all autologous techniques entail some degree of donor-side morbidity, requiring a secondary procedure for tissue removal [60], studies indicate that sural nerve harvest is well-tolerated. This is attributed to the fact that the sural nerve supplies a relatively insignificant dermatome, contributing to the overall acceptability of this donor site [60, 62].

In cases where autologous nerves are unavailable for harvest or the patient declines this option, autologous vein grafts present an alternative for nerve bridging. Unlike autologous nerve grafts, these vein grafts primarily function as conduits to bridge the defect. The vein graft is often inverted, with the expectation that the growth factors on the external side will positively influence nerve regeneration [63]. Nevertheless, veins are reported to have a lower success rate compared to nerves, with rates of 87% for nerves versus 60% for veins [1, 51]. This difference underscores the variations in effectiveness between these two grafting options, with nerves generally exhibiting a higher success rate in promoting successful nerve regeneration.

#### Allografts, alloplastic and xenogeneic alternatives

To mitigate donor-side morbidity, there is ongoing research on processed-decellularized allografts [47]. These allografts demonstrate high success rates, exceeding 85%, in achieving sensory restoration for both smaller and larger defects (documented in the literature for defects up to 7 cm [61]), albeit in limited patient numbers thus far [48, 61, 64]. In addition to autologous and allogeneic grafts, various alloplastic or xenogeneic scaffolds are available, which can be either resorbable or non-resorbable. These are primarily utilized for smaller defects, up to 2–3 cm in size, and are composed of materials such as polyglycolic acid [65, 66], poly-(DL-lactide-co-caprolactone) [67], type 1 collagen [68, 69], or chitosan [70].

### Conservative treatment

While negative symptoms can be distressing for the patient and may pose challenges in daily life, they are typically not amenable to medication influence. On the other hand, positive symptoms, such as neuropathic pain, often necessitate specific treatment. Realistic objectives of medical therapy for neuropathic pain include achieving a pain reduction of ≥ 30%, enhancing sleep and overall quality of life, improving functionality, maintaining social activity and relationship structures, and preserving the ability to work [18]. At the onset of such therapy, it is crucial to inform the patient about potential side effects and the possibility of a delayed onset after dosing to enhance compliance. The effectiveness of a combination of several drugs has been demonstrated in managing neuropathic pain [18, 71].

It is important to note that not all products listed below are approved for treating neuropathic pain and are, therefore, used off-label. Schlereth et al. summarize in their guideline that a drug for off-label use should have proven efficacy with a favorable benefit-risk profile and a lack of alternatives. Additionally, the physician must inform the patient, especially about potential consequences, including the lack of liability on the part of the manufacturer [18].

#### Topical medication

The use of a topical local anesthetic, such as 5% lidocaine or 8% capsaicin, may be beneficial for neuralgic pain. However, the available studies primarily pertain to application in the form of a plaster, which could pose challenges for intraoral use. Lidocaine, known for its voltage-gated sodium channel blocking effect, is recommended at a dosage of 1–3 patches (each containing 700 mg lidocaine [72]) for application to dry, intact, and non-irritated skin over 12 h, followed by a mandatory 12-h application-free interval. Given that approval is limited to post-zoster neuralgia, employing this treatment for post-traumatic neuralgia of the trigeminal nerve constitutes an off-label use [18]. Despite being an off-label use, initial promising studies have emerged, indicating successful treatment outcomes for trigeminal neuralgia [73, 74], Hence, 5% lidocaine plasters may be considered as an interesting pharmacological option. The utilization of lidocaine plasters is deemed safe, with an anticipation of minimal side effects, limited to local skin reactions, and no anticipated interactions with other medications.

Capsaicin binds to the TRPV1 channel (transient receptor potential) and, consequently, stimulates nociceptors, which are receptors that detect pain stimuli associated with heat or chemical irritation. This stimulation leads to reactive hyperemia and the release of endorphins. The existing literature consistently indicates a reduction in neuropathic pain following the application of 8% capsaicin plasters (each patch containing 179 mg capsaicin). The recommended application is for a maximum of 60 min, with a limit of four plasters simultaneously. In certain cases, this effect surpasses the efficacy of pregabalin and gabapentin and is comparable to that of duloxetine [49, 75]. Furthermore, successful utilization in cases of traumatic trigeminal neuropathies has been documented [76]. Capsaicin plasters are sanctioned for the management of peripheral neuropathic pain in adults. Both 5% lidocaine and 8% capsaicin are consequently favored, especially in cases of focal nerve lesions, owing to their reduced side effects compared to systemic agents [14, 18, 76].

#### Botulinum toxin

Emerging evidence suggests that botulinum toxin A (BTX) may exert notable analgesic effects with minimal side effects in both traumatic and nontraumatic neuropathy, including posttraumatic neuralgia of the trigeminal nerve [77–81]. The inhibition of acetylcholine release by BTX results in the suppression of nerve excitation transmission. Beyond its paralytic effects, recent studies propose an analgesic impact achieved by blocking the release of algogenic neuropeptides, including substance P, neurokinin A, and calcitonin gene-related peptide, as well as glutamate, in primary small-diameter type C afferent nerve fibers [79, 82]. The recommended dosage for injection near the nerve trunk [81] appears to be in the range of 50–200 IU, despite the lack of current approval for this indication. Schlereth et al. suggest that BTX may be considered for treating neuropathic pain of any origin; however, it is designated as a third-line drug for addressing focal limited symptoms [18, 49].

#### Systemic corticosteroids

In the initial stages of suspected nerve damage, especially in cases of neurapraxia, anti-inflammatory drugs may prove beneficial. This is particularly applicable to high-dose oral corticosteroids, such as 25 mg prednisolone twice daily, within the first ten days [83, 84]. Nevertheless, the evidence supporting such treatment is limited, although corticosteroids are frequently used in neurosurgery and have been shown to enhance the outcomes of facial nerve palsies [84]. The proposed mechanism of action involves the modulation of the immune response or a direct reduction of perineural edema. However, the use of corticosteroids can lead to significant side effects, particularly with prolonged and high-dose administration. If the patient does not respond to the treatment, discontinuation should be considered after seven to ten days [85, 86]. The discontinuation of corticosteroids should be gradual, taking into account the duration of therapy, dosage, and other factors, such as repeated therapy.

#### Non-opioid analgesics

There is no evidence of efficacy for the use of non-opioid analgesics such as Cyclooxygenase inhibitors, acetaminophen, and metamizole in neuropathic pain [18]. Hence, they should not be employed in this condition [87, 88].

#### Co-analgesics

If the injury persists for an extended period, treatment options such as tricyclic antidepressants, anticonvulsants affecting neuronal calcium channels, serotonin-norepinephrine reuptake inhibitors (SNRIs), selective serotonin reuptake inhibitors (SSRIs), and surgery, or a combination of these approaches, are considered promising. However, it is important to note that these drugs may have significant side effects that could limit their long-term use [89].

#### Antidepressants

##### Tricyclic antidepressants (TCAs)

Amitriptyline, imipramine, and clomipramine function primarily as serotonin and norepinephrine reuptake inhibitors through transporter blockage, leading to increased synaptic concentrations and enhanced neurotransmission. Additionally, these medications affect various other receptors, including the H1 histamine receptor, alpha1 adrenoreceptor, and muscarinic acetylcholine receptor, potentially resulting in associated side effects [18, 90]. Tricyclic antidepressants (TCAs), with amitriptyline being the most commonly used TCA, are recommended as first-line medications for the treatment of neuropathic pain of any cause. Amitriptyline is approved for neuropathic pain treatment in adults, while imipramine and clomipramine are approved for long-term pain treatment as part of an integrated plan. Individualized titration is necessary, considering potential side effects. Amitriptyline therapy, for instance, typically begins at a low dose (10–25 mg at night) and is gradually increased, with the daily dose raised by 10 to 25 mg every three to seven days [18, 90]. The analgesic dosage of TCAs, typically ranging from 25 to 75 mg daily, is usually below the antidepressant dosage (Table 1). The analgesic effect usually manifests with a treatment delay of 2–4 weeks. Common side effects of TCAs include dry mouth, constipation, weight gain, erectile and micturition problems, and hypotonic circulatory disturbances. Multiple drug interactions must also be considered when using TCAs. Amitriptyline, in particular, exerts a sedative effect (H1 antagonism), which may be beneficial in addressing sleep disturbances associated with neuropathic pain [18]. Elderly patients (> 65 years of age) should ideally avoid TCAs due to potential cardiac complications and significant anticholinergic effects. Accordingly, TCAs are considered unsuitable for elderly patients.

**Table 1:** Dosing of antidepressants as co-analgesics

Substance	Initial dosage/day	Target dosage/day
Amitriptylin	10–25 mg	25–75 mg
Duloxetin	20–30 mg	60–120 mg

##### Selective serotonin-norepinephrine reuptake inhibitors (SNRIs)

SNRIs primarily function by augmenting the descending inhibitory pain processing system, leading to increased availability of norepinephrine and serotonin, with norepinephrine playing a crucial role [91]. Analgesia occurs independently of the antidepressant effect, typically even at low doses. While the majority of data pertains to duloxetine, it's worth noting that studies on chronic pain and SNRIs are predominantly focused on diabetic neuropathy [91]. Duloxetine is currently approved solely for the treatment of diabetic polyneuropathy, depressive disorders, and generalized anxiety disorders. Its use in the treatment of neuropathic pain, while considered off-label, is increasingly recommended as a first-line option [18]. A recent extensive meta-analysis, evaluating the efficacy and safety of antidepressants in chronic pain patients, encompassing nearly 30,000 participants across 176 studies, revealed that duloxetine exhibited the most favorable efficacy. A standard dose of 60 mg was found to be effective, providing approximately 50% pain relief in 435 out of 1,000 subjects, compared to 287 subjects in the placebo group. However, the review highlighted a lack of reliable evidence regarding the long-term efficacy and safety of antidepressants in chronic pain, emphasizing gaps in the current literature [92].

Typically, duloxetine treatment initiates at 30 mg, progressing to a target dose of 60 mg within one to two weeks (maximum dose 120 mg). After two months, an evaluation of effectiveness is recommended, as further increases beyond this period may offer limited benefits for patients with an unsatisfactory initial response. Common side effects may encompass initial nausea and vomiting, headache, xerostomia, fatigue, and dizziness. However, individual responses to side effects vary, and it is crucial to consider contraindications and relevant drug interactions [18, 93]. For instance, it is important to note that duloxetine may heighten the risk of bleeding in patients using oral anticoagulation. Nonetheless, the superior tolerability of SRNIs in comparison to TCAs positions them as a favorable choice for managing neuropathic pain (Table 1).

#### Anticonvulsants acting on neuronal calcium channels

Gabapentin and pregabalin act by binding to presynaptic voltage-gated calcium channels in the posterior horn of the spinal cord. This binding leads to a reduction in the release of excitatory neurotransmitters, including glutamate and substance P [94]. As per current guidelines, both gabapentin and pregabalin are approved for the management of peripheral and central neuropathic pain. They are recommended as first-line medications for the treatment of chronic neuropathic pain [18]. This recommendation pertains to a daily dose range of 1200–3600 mg for gabapentin and 300–600 mg for pregabalin [18, 95] (Table 2). Reported side effects of gabapentin and pregabalin include drowsiness, dizziness, peripheral edema, visual disturbances, gait disturbances, and ataxia [50, 96].

**Table 2:** Antiepileptic drugs as co-analgesics

Substance	Initial dosage/day	Target dosage/day
Gabapentin	2 × 100–300 mg	3 × 300–1200 mg (< 2400 mg)
Pregabalin	2 × 75 mg	2 × 150–300 mg (< 600 mg)

Pregabalin, especially with drug dependency and high daily doses exceeding the maximum of 600 mg, possesses a strong potential for addiction (median > 2000 mg). In isolated cases, life-threatening intoxications have been reported in cases of poly-drug consumption (benzodiazepines, alcohol, other drugs). Pregabalin should be avoided in patients with pre-existing drug dependency, and if therapy is required, the patient should be monitored closely for any abuse.

Given the favorable pharmacokinetics and lower toxicity in oral overdose, gabapentin should be considered in cases of potential addiction. The poor oral absorption and nonlinear pharmacokinetics characteristic of gabapentin are problematic. Absorption and transition to the central nervous system involve a saturable transporter. Varying individually, the effective fraction no longer increases proportionally when higher doses are administered. Longer adjustment phases are, therefore, often necessary to determine the correct dosage. Both substances, gabapentin, and pregabalin, should always be started and stopped gradually. A therapeutic attempt with the respective other drug is useful if the first substance is ineffective.

Gabapentin and pregabalin are considered first-line agents for neuropathic pain. However, it is challenging to predict which drug will result in symptom improvement for a particular patient [18, 95]. Both agents offer the advantage of not undergoing hepatic metabolism and having no known drug interactions, unlike other antiepileptic drugs. Common side effects include drowsiness, headache, edema, and nausea. Dose adjustment is necessary for patients with impaired renal function.

#### Opioids

Opioids function as ligands at opioid receptors, exerting a primarily central analgesic effect. Generally, opioids are recommended as second- to third-line therapy for the treatment of neuropathic pain [18, 50, 95]. The principal limiting factors include the significant potential for abuse and an escalating mortality rate attributed to overdoses, notably observed in the USA, Canada, and Great Britain. Insufficient data are available regarding overall effectiveness. Alongside the risk of concurrent addictive disorders and the development of tolerance, extended use of opioids commonly leads to somnolence, sedation, constipation, and nausea. A comprehensive risk–benefit analysis is imperative when considering the utilization of opioids. A large cohort study has documented challenges in daily functioning, depression, and impaired occupational capabilities during prolonged therapy.

## Conclusion

In summary, this scoping review endeavors to provide valuable insights for dentists and practitioners engaged in the diagnosis and treatment of patients affected by traumatic injuries to the inferior alveolar and lingual nerves. While the current literature presents limitations in terms of evidence, this compilation serves as a comprehensive guide based on the existing knowledge. However, it is crucial to acknowledge a major limitation in the form of the absence of a systematic approach to this review, which might impact the precision and robustness of the presented information.

Moving forward, the imperative for the dental community lies in the initiation of prospective randomized controlled trials. These studies are essential to rigorously investigate and validate the diagnostic and therapeutic strategies outlined in this review. The implementation of such trials holds the promise of establishing official evidence-based guidelines, refining clinical practices, and enhancing the overall quality of care provided to individuals experiencing traumatic injuries to the inferior alveolar and lingual nerves. As the field advances, a commitment to scientific rigor and a collaborative effort within the dental research community will be vital to address the existing gaps and ensure the continual improvement of diagnostic and treatment protocols in this specialized area.

## Data Availability

No datasets were generated or analysed during the current study.

## Abbreviations

BTX: Botulinum toxin A
DFNS: German Neuropathic Pain Research Network
QualST: Qualitative sensory test
QST: Quantitative sensory test
SSRI: Selective serotonin reuptake inhibitor
SNRI: Serotonin-norepinephrine reuptake inhibitor
TCA: Tricyclic antidepressants
